# Mosquito Microbiomes of Rwanda: Characterizing Mosquito Host and Microbial Communities in the Land of a Thousand Hills

**DOI:** 10.1007/s00248-024-02382-3

**Published:** 2024-05-01

**Authors:** Amanda G. Tokash-Peters, Jean Damascene Niyonzima, Mirielle Kayirangwa, Simon Muhayimana, Ivan W. Tokash, Jaimy D. Jabon, Sergio G. Lopez, Patrick J. Kearns, Douglas C. Woodhams

**Affiliations:** 1 College of Science and Mathematics, University of Massachusetts Boston, Boston, MA USA; 2 Center of Excellence in Biodiversity, University of Rwanda, Huye, Rwanda

**Keywords:** Rwanda, Mosquito microbiome, Vector-borne disease, Disease ecology

## Abstract

Mosquitoes are a complex nuisance around the world and tropical countries bear the brunt of the burden of mosquito-borne diseases. Rwanda has had success in reducing malaria and some arboviral diseases over the last few years, but still faces challenges to elimination. By building our understanding of in situ mosquito communities in Rwanda at a disturbed, human-occupied site and at a natural, preserved site, we can build our understanding of natural mosquito microbiomes toward the goal of implementing novel microbial control methods. Here, we examined the composition of collected mosquitoes and their microbiomes at two diverse sites using Cytochrome c Oxidase I sequencing and 16S V4 high-throughput sequencing. The majority (36 of 40 species) of mosquitoes captured and characterized in this study are the first-known record of their species for Rwanda but have been characterized in other nations in East Africa. We found significant differences among mosquito genera and among species, but not between mosquito sexes or catch method. Bacteria of interest for arbovirus control, *Asaia*, *Serratia*, and *Wolbachia*, were found in abundance at both sites and varied greatly by species.

**Supplementary Information:**

The online version contains supplementary material available at 10.1007/s00248-024-02382-3.

## Introduction

Mosquito-borne diseases disproportionately impact nations in the tropics, with Sub-Saharan Africa bearing a sizable burden [48, 61]. Annually around the world, there are between 100 and 400 million cases of Dengue fever, 200,000 cases of yellow fever, 249 million cases of malaria, and several hundred periodic cases of Rift Valley fever [6, 10, 14, 21, 38, 50, 61–63]. While some control efforts have stagnated over time, Rwanda has effectively reduced the number of malaria cases by 3.8 million from 2020 to 2022 [27, 63].

Despite this remarkable reduction, challenges still exist in further reducing arbovirus and parasite transmission. One mode of control that shows potential in pushing toward reduction goals is microbial manipulation of mosquito hosts [7, 15, 19, 24, 32, 35, 54, 56, 58, 59]. Introduction of bacteria of the genera *Asaia*, *Serratia*, and *Wolbachia* into the mosquito microbiome have all been implicated as potential methods for pathogen and parasite control. These bacteria can either trigger an immune response in the mosquito that can help reduce prevalence of pathogens and parasites or can directly compete with invading microbes [29, 23, 28, 46, 51, 56, 58]. Though the World Mosquito Program has begun implementing the release of *Wolbachia*-infected *Aedes* mosquitoes with success, there are still numerous uncertainties regarding in situ release and efficacy of these methods [25, 36, 42, 64].

In order to better understand the probability of success for microbial methods of control in East Africa, a background understanding of naturally formed mosquito host and microbiome communities is needed. While we are still building our understanding of factors that influence the formation of mosquito microbiomes, we know that the environment plays an important role and is scale dependent [2, 13, 18, 57].

In this study, we aimed to characterize the mosquito and microbial communities of two sites in Rwanda. We hypothesized that there would be differences in microbial composition among mosquito taxa, mosquito sexes, and whether mosquito females were visibly blood-fed or not. Future studies may better characterize regional differences using this study as a baseline description of mosquito communities and establishment of novel records for mosquitoes in Rwanda.

## Methods

### Site Selection and Research Permit

Two sampling sites in Rwanda were selected for characterization of mosquito microbiomes. Samples were collected in February 2019 between the peak dry and wet seasons. Sites were selected that had nearby (< 500 m) frequently traversed walking paths. Sites were also secondary growth forests with similar canopy and ground cover. Site 1 (Nyabugogo disturbed) was near Kigali, upland of Nyabugogo marsh, and in a grove of trees in a highly disturbed area with densely packed temporary housing with poor sanitation conditions. Site 2 (Huye campus) was the arboretum on the Huye campus of the University of Rwanda which contains a footpath frequently traversed by members of the community but no settlements in the immediate vicinity of the site.

### Ethics Statement

Field sampling was performed under the authority of the Rwandan National Council for Science and Technology (NCST) under Permit # NCST/482/62/2018.

### Specimen Collection, Storage, and Extraction

Mosquito samples were collected by kicking through the brush along the transect away from the trail and catching mosquitoes that emerged from the brush. These were then caught by gloved hand or by aspirator for a 3-h period at sunrise. A new pair of gloves was used between specimens or cleaned with 70% ethanol between grabs or aspirator collections. Additional samples at each site were collected while resting on surrounding foliage using either aspirator or hand catch with gloves. Samples were frozen at − 20 °C upon collection until they could be processed, and then had the head and legs removed before being homogenized. Samples were individually stored in 1X DNA/RNA Shield (Zymo Research) and were extracted using Quick DNA/RNA MagBead kits (Zymo Research) and stored at − 80 °C.

### Cytochrome C Oxidase Subunit I Barcoding

Mosquito species identification was performed using cytochrome c oxidase subunit I conserved gene barcoding. PCR was performed using the standard LCO1490 and HCO2198 primers to target a 710-bp fragment [20]. Polymerase chain reaction (PCR) was executed in duplicate using 10 µL 5Prime HotMasterMix (Quantabio), 11 µL PCR grade water, 1 µL 10 uM LCO1490), 1 µL 10 uM HCO2198, and 2 µL template DNA [20]. The PCR cycling conditions were 94 °C for 3 min, 35 cycles of 94 °C for 45 s, 50 °C for 60 s, and 72 °C for 90 s, followed by 72 °C for 10 min. Samples were then pooled and sent to MC Lab (South San Francisco, CA, USA) for PCR clean-up and sequencing using an ABI 3730XL sequencer. Upon retrieval of sequences from MC Lab, FASTA sequences were matched to mosquito species-level identifications using the BOLD Systems Identification Engine for all records on BOLD as of March 2020 [47]. The highest percentage match at the species level of identification was used as the positive identification of the sample. Samples with exceptionally poor species level matches (< 85%) were not included in further analyses utilizing the taxonomic identification. To ensure that identification of each species would be highly likely in Rwanda, species were all cross-referenced in BOLD, the Walter Reed Biosystematics Unit Systematic Catalogue of Culicidae, and the broader literature for known records of presence in East Africa (see the Supplementary information). Identified species of mosquitoes were broadly classified into generalized habitat use groups (Domestic, Sylvatic, Ubiquitous, Unknown, or Wetland) based on existing literature of habitat use, particularly in East Africa whenever possible (see references in the Supplementary information).

### Microbiome Sequencing and Preparation

The 16S rRNA gene V4 region was amplified by PCR using the standard 515F and 806R primers with barcodes on the forward primer consistent with the Earth Microbiome Project (EMP) protocols [4, 12, 13, 44, 55]. PCR was performed with minor modifications to the EMP protocols, amplifying samples and controls (including extraction controls and PCR controls) in duplicate using 10 µL 5Prime HotMasterMix (Quantabio), 8.5 µL PCR grade water, 3.5 µL 10 uM 806R, 3.5 µL 10 uM barcoded 515F, and 2 µL template DNA. Cycling conditions followed the standard EMP protocol and can also be found listed above in the methods section on COI barcoding. PCR-amplified samples were then normalized using the Mag-Bind Pure Library Normalization Kit (Omega Bio-Tek) and pooled for library quantification and subsequent dilution using a Qubit 2 Fluorometer (Invitrogen). PhiX was added according to recommendations from the Earth Microbiome Project and the library was sequenced using an Illumina MiSeq v2 300-cycle kit at the University of Massachusetts Boston [52, 53].

Sequencing adaptors were removed prior to fastq generation on the sequencer. Due to the OTU picking method, we analyzed only the first read generated by the sequencer. Reads were quality filtered with a minimum quality score of 20 and reads with ambiguous bases were removed. Reads were truncated after three low quality bases and we kept sequences that were at least 80% of the original read length of 151 bp. Reads were trimmed to 120 bp and deblur was used to resolve sub-operational taxonomic units (sOTUs; [3]). A phylogenetic tree was generated with fasttree [45] and taxonomy was assigned to sOTUs with sklearn [40] using the GreenGenes database as a reference (version 13.8; [17]).

### Bioinformatic and Statistical Analyses

**Fig. 1:**
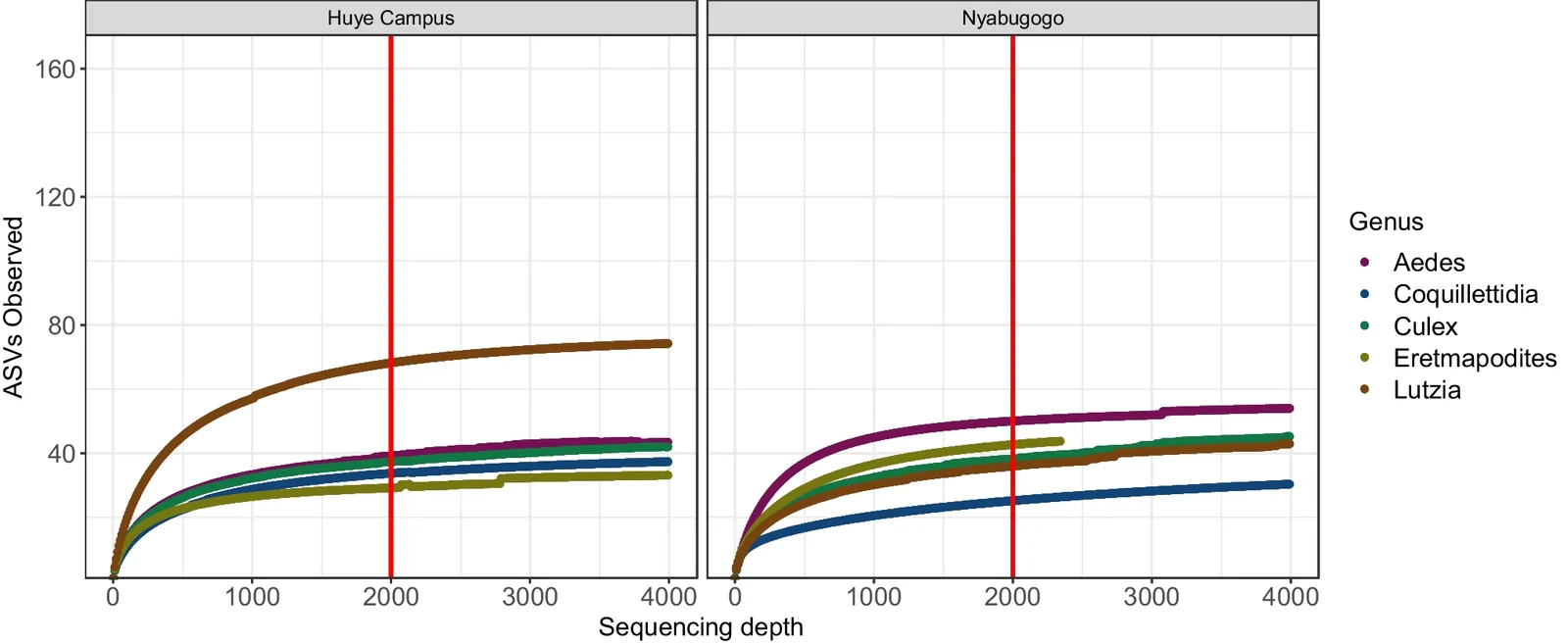
Rarefaction curves of bacterial amplicon sequence variants (ASVs) from mosquito genera at two sites in Rwanda. Line at 2000 reads indicates rarefaction depth chosen for inclusion of most samples for analysis representing the majority of ASVs

Analysis of mosquito microbiomes were performed using QIIME2 and the R packages vegan, MicrobeR, and qiime2r [8, 9, 41]. Samples were rarefied to 2000 sequences per sample in QIIME2 as this depth rarefaction curves plateaued (Fig. 1). Kruskal–Wallis pairwise tests were performed to analyze differences in sOTU richness and Shannon Diversity between experimental groups. PERMANOVAs were performed to assess statistical differences in the beta diversity of microbial communities between experimental groups using the PERMANOVA function in QIIME2. PERMANOVAs were performed on both Jaccard and unweighted UniFrac [31] distance matrices with 999 permutations. We found no significant differences in metrics (Mantel test, R = 0.89, *p* < 0.01), thus we only report unweighted UniFrac distances. Finally, we analyzed the core microbiome by examining the distribution and abundance of the top twenty sOTUs across all samples.

## Results

### Mosquito Community Results

Between both the Nyabugogo disturbed and Huye campus sites, 447 individuals belonging to forty species of mosquitoes were successfully identified across six genera (Table 1). Female captures greatly outnumbered male captures, at approximately double the rate across both sampling sites. Based on BOLD and the Walter Reed Biosystematics Unit Systematic Catalogue of Culicidae, 36 of the 40 species collected were potentially the first record of these mosquitoes in Rwanda when they were collected in 2019, though nearly all of these species were found in other east African nations. *Culex* was the most prevalent genus of mosquitoes at both sites. *Aedes* mosquitoes were highly prevalent at the Huye campus site and *Lutzia* mosquitoes were common at the Nyabugogo disturbed site. Overall, *Lutzia tigripes*, *Culex univitattus*, and *Culex decens* were the most frequently captured species. At the Huye campus site, *Culex univitattus, Culex decens*, and *Aedes mcintoshi* were the most frequently encountered species, while *Lutzia tigripes*, *Culex univitattus*, and *Culex striatipes* were the most encountered at the Nyabugogo disturbed site. (See the Supplementary information, mapping file for complete identification and site information.)

**Table 1:**
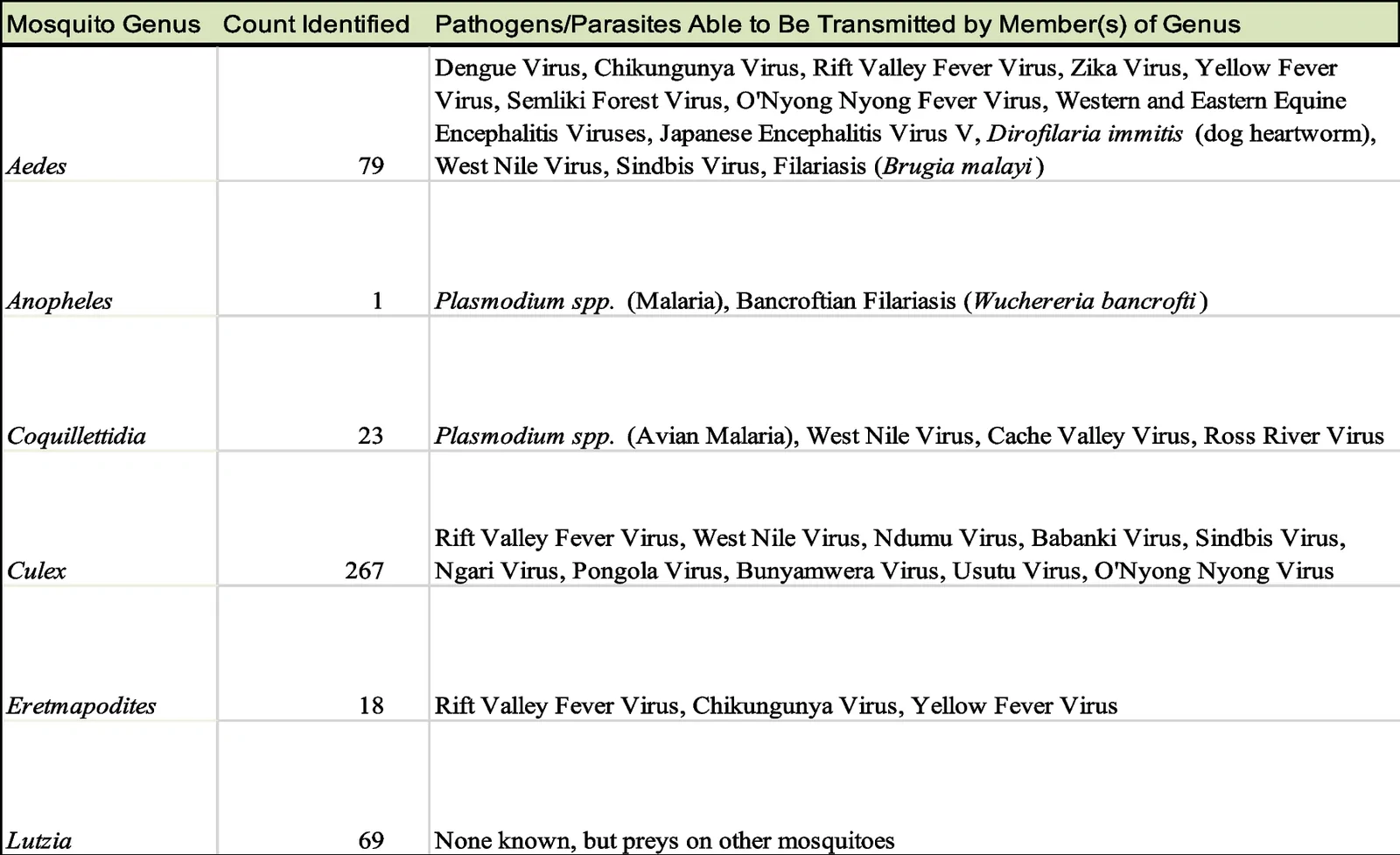
Mosquitoes sampled in Rwanda in this study and classified using cytochrome c oxidase subunit I (COI) gene sequencing. Pathogens and parasites that each genus is capable of transmitting are listed. No viruses were directly characterized as being present in any of the collected samples and this list is not intended to be exhaustive. See Supplementary information for a list of references

**Fig. 2:**
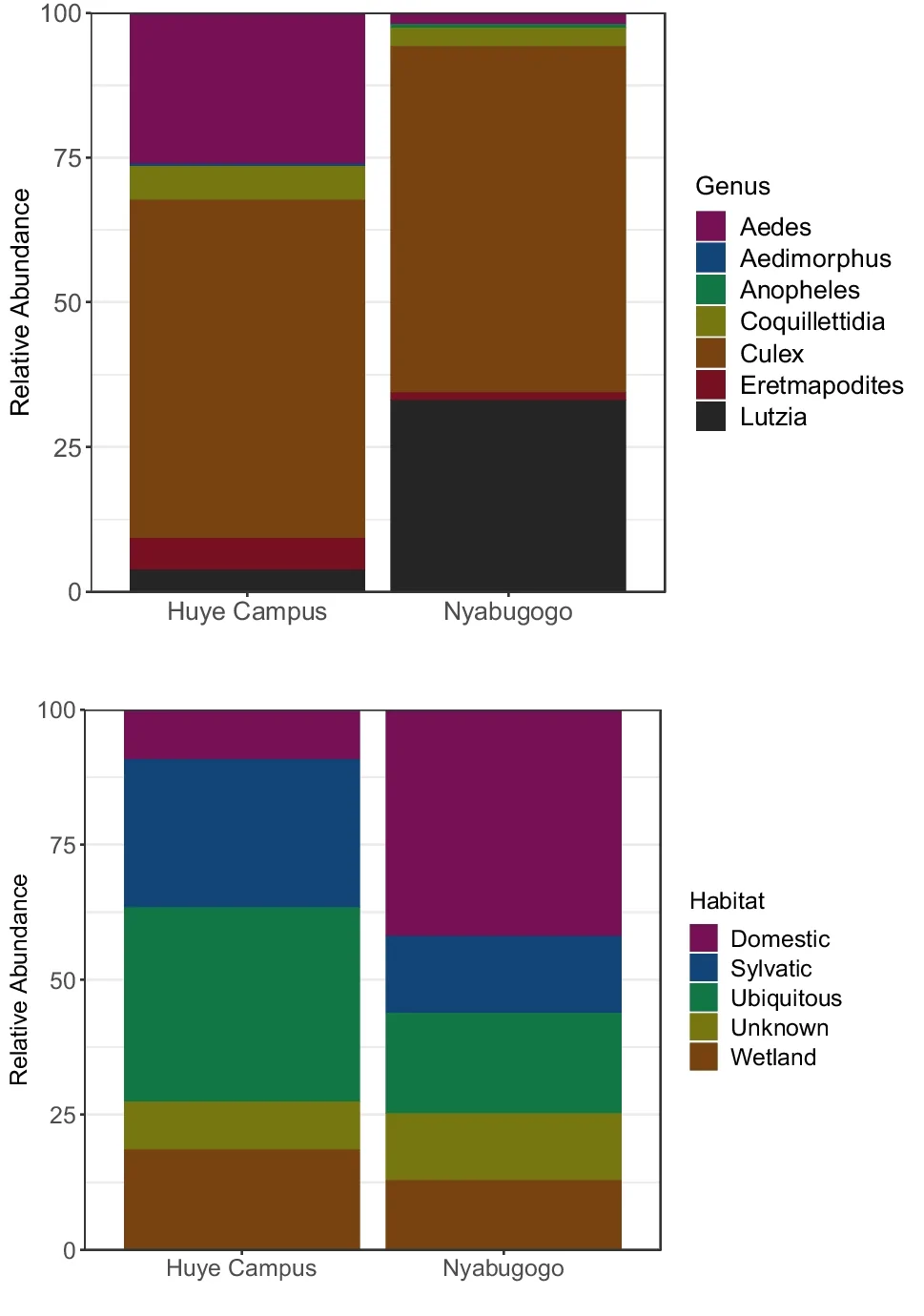
Stacked barcharts comparing the relative abundance and assemblage of mosquitoes captured at the Huye Campus (arboretum) and Nyabugogo (highly disturbed) sites (A, upper panel), and relative abundance in percentage of all mosquitoes by sampling site for each generalized habitat-use categorization (B, lower panel)

The community composition of mosquitos differed substantially between sites (Fig. 2). The Huye Campus site had greater numbers of *Aedes* sp., *Coquilletidia* sp., and *Eretmapodites* sp., whereas Nyabugogo had more *Lutzia* sp. and accounted for the single *Anopheles* sp. mosquito collected. *Culex* sp. mosquitoes were the majority of mosquitoes at both sites, though the species composition of *Culex* sp. differed.

**Fig. 3:**
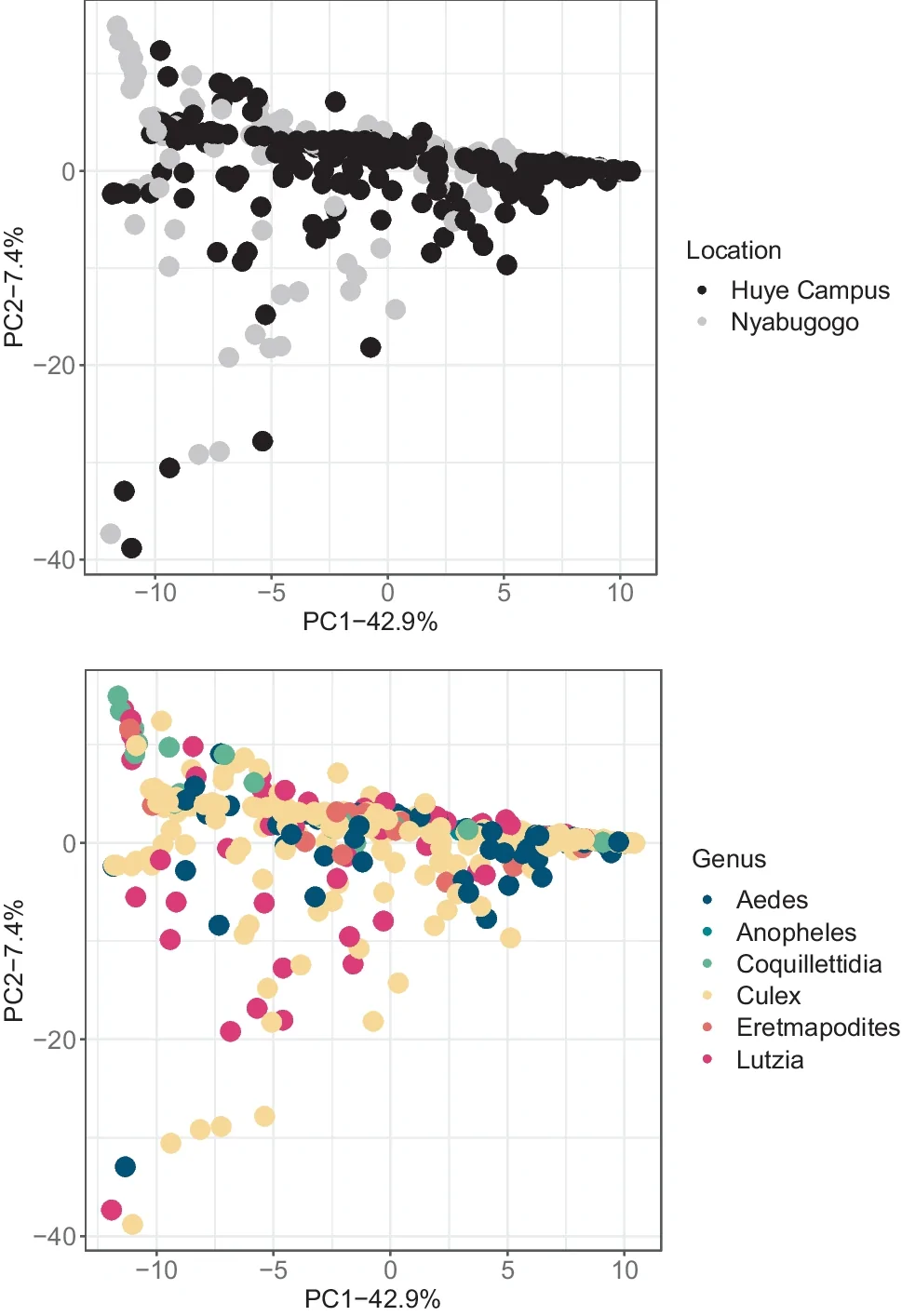
Principal coordinate analysis based on Bray–Curtis distances to demonstrate the relative community composition of the bacterial microbiome by sampling location (A, upper panel), and for each genus (B, lower panel). Axes indicate percent variation explained

In terms of the generalized habitat use assignments, mosquitoes classified as ubiquitous were the most abundant overall, in particular at the Huye campus site (Fig. 3). At the Nyabugogo disturbed site, domestic mosquitoes were most common and nearly double the number of ubiquitous mosquitoes captured. Sylvatic mosquitoes were highly abundant at the Huye campus site, while they were far less prevalent at the Nyabugogo disturbed site. Specifically in terms of relative abundance, there were more wetland mosquitoes at the Huye campus site than at the Nyabugogo disturbed site.

### Microbiome Diversity

#### Alpha Diversity of the Microbiome

Visibly blood fed and non-blood fed mosquitoes were marginally different in terms of microbiome sOTU richness (*p* = 0.06) but not Shannon Diversity (p > 0.05) in a pairwise Kruskal–Wallis test using (*p* = 0.06). There was no other significant difference between any groups in terms of microbiome sOTU richness or Shannon Diversity, including sex, location, catch method, collection date, or processing date.

#### Beta Diversity of the Microbiome

There were significant differences when tested using PERMANOVAs between weighted Unifrac values for sampling locations (*n* = 442, pseudo-F = 9.02676, *p* = 0.001), genera (*n* = 466, pseudo-Fc = 2.48844, p = 0.002), species (*n* = 466, pseudo-F = 2.01142, *p* = 0.001), catch dates (*n* = 466, pseudo-F = 5.16716, *p* = 0.001), and processing dates (*n* = 466, pseudo-F = 2.97262, *p* = 0.001). As only one site was sampled per day and which groups of mosquitoes were processed on which date was non-random, these were considered to be an artifact of these variables strongly co-varying with location. Mosquito sexes (*n* = 454, pseudo-F = 2.02918, *p* = 0.068), visibly blood-fed status (*n* = 466, pseudo-F = 0.964349, *p* = 0.457), and catch methods (*n* = 466, pseudo-F = 1.53627, *p* = 0.115) were all not significantly different. Principal coordinate analysis (Fig. 3) demonstrates the differences in community composition of the mosquito genera at both sampling locations. *Aedes* and *Coquillettidia* from the Huye campus site have visually high dispersion in their microbial community compositions compared to those genera at the Nyabugogo disturbed site. *Lutzia* has higher dispersion at the Nyabugogo disturbed site, while *Culex* is fairly disperse at both sites (Fig. 3). Principal coordinate analysis based on weighted Unifrac distances demonstrate the relative community composition of the microbiome for each species by sampling location (Supplementary information, Figure S1).

#### The Core Microbiome

**Fig. 4:**
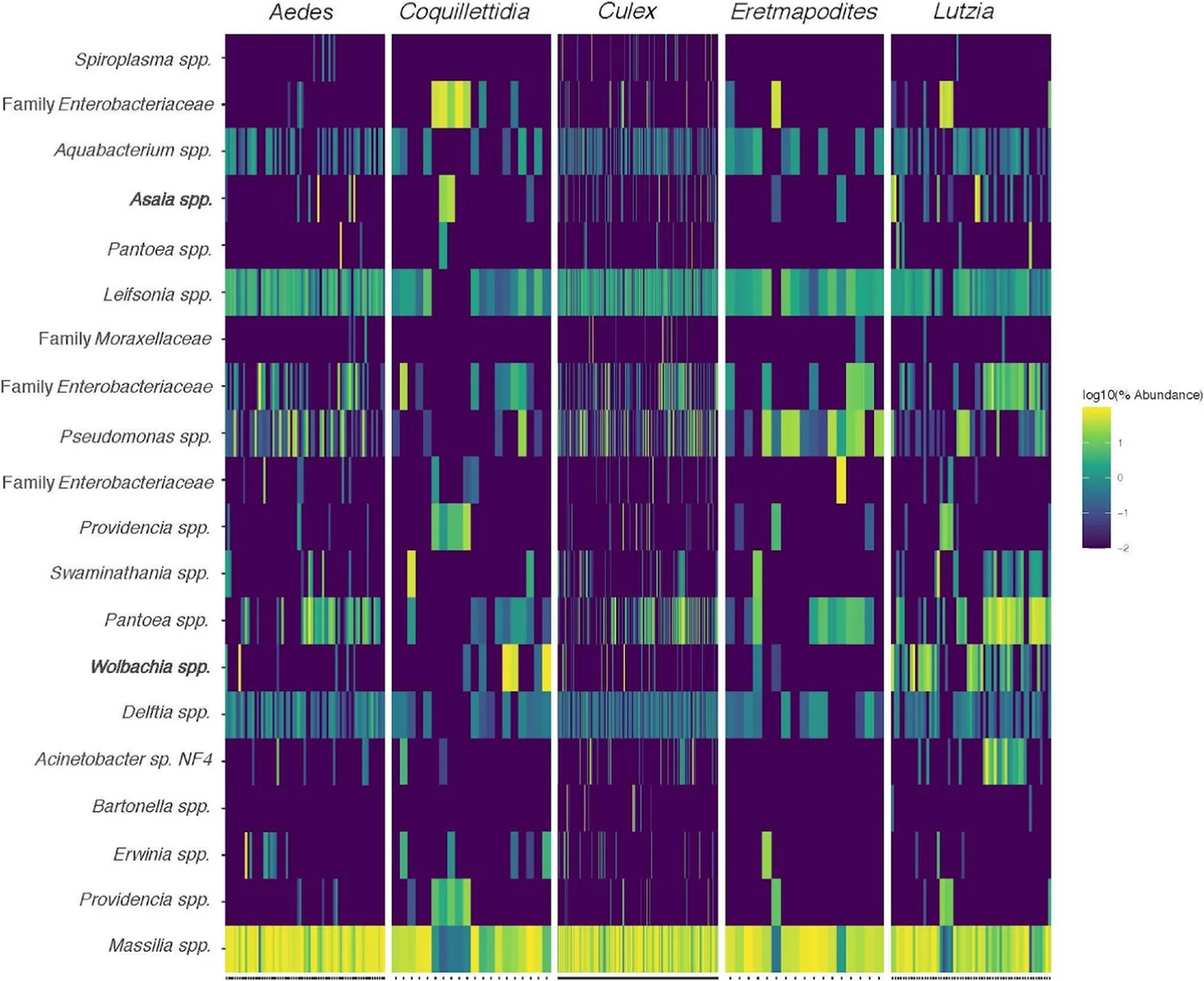
Heatmap of the 20 most abundant sOTUs across all mosquito samples grouped by genus. *Massilia* spp. was the most overall abundant sOTU, with high abundances of *Leifsonia* spp., *Delftia* spp., *Pantoea* spp., and *Pseudomonas* spp. Other notable members of the core microbiome include *Wolbachia* spp. and *Asaia* spp. that have been noted for their role in the control of arboviral transmission

Core members of the mosquito microbiome were considered to be the top 20 most abundant sOTUs across all mosquito samples (Fig. 4). The most abundant sOTUs were *Massillia* spp., *Leifsonia* spp., and *Delftia* spp. Other notable members of the core microbiome include *Wolbachia* spp. and *Asaia* spp.

#### Wolbachia and Bacteria of Anti-arboviral Interest

**Fig. 5:**
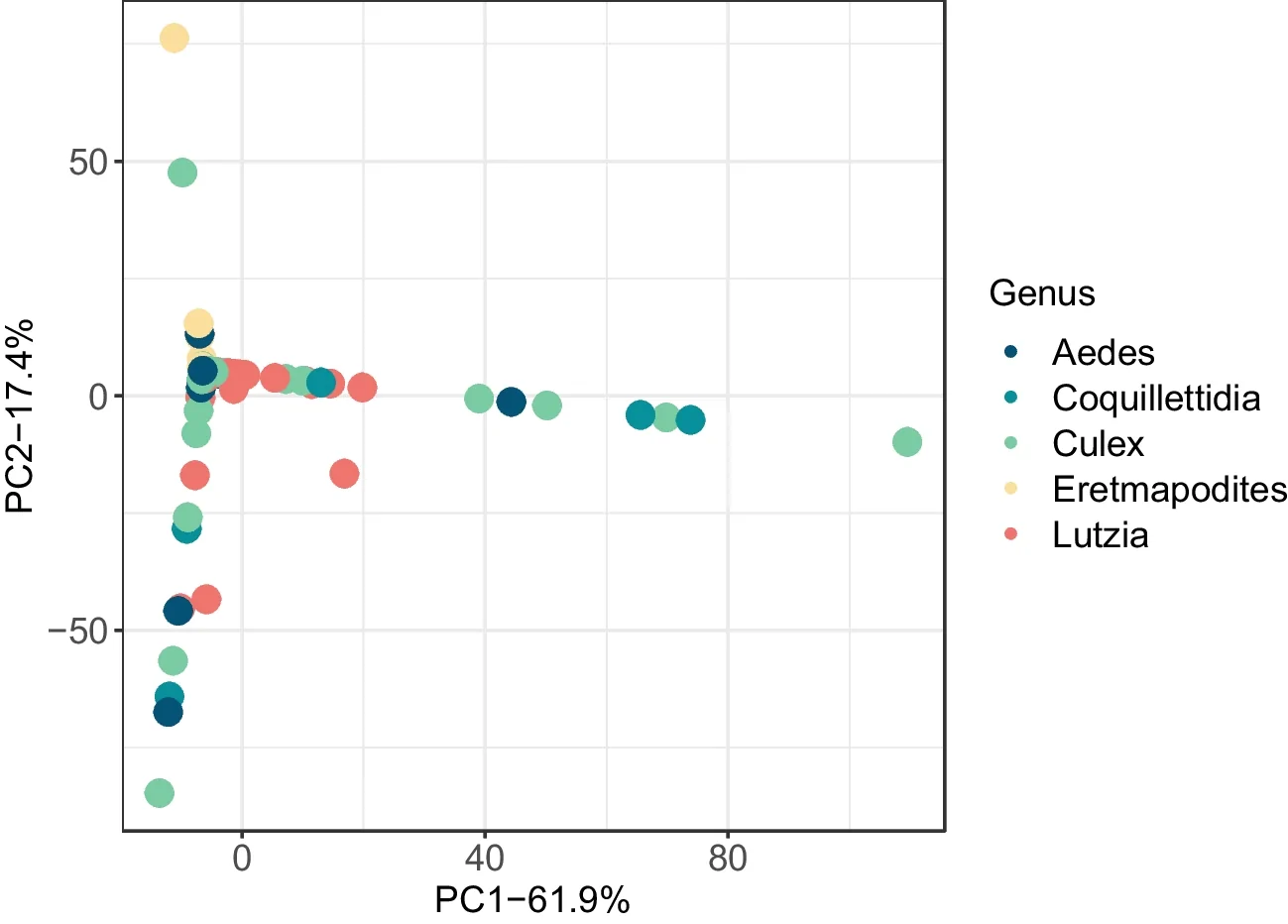
Principal coordinate analysis based on Bray–Curtis distances to compare among mosquito genera the relative community composition of the microbiome component thought to contribute to arboviral transmission dynamics (includes only *Asaia*, *Serratia*, and *Wolbachia*)

**Fig. 6:**
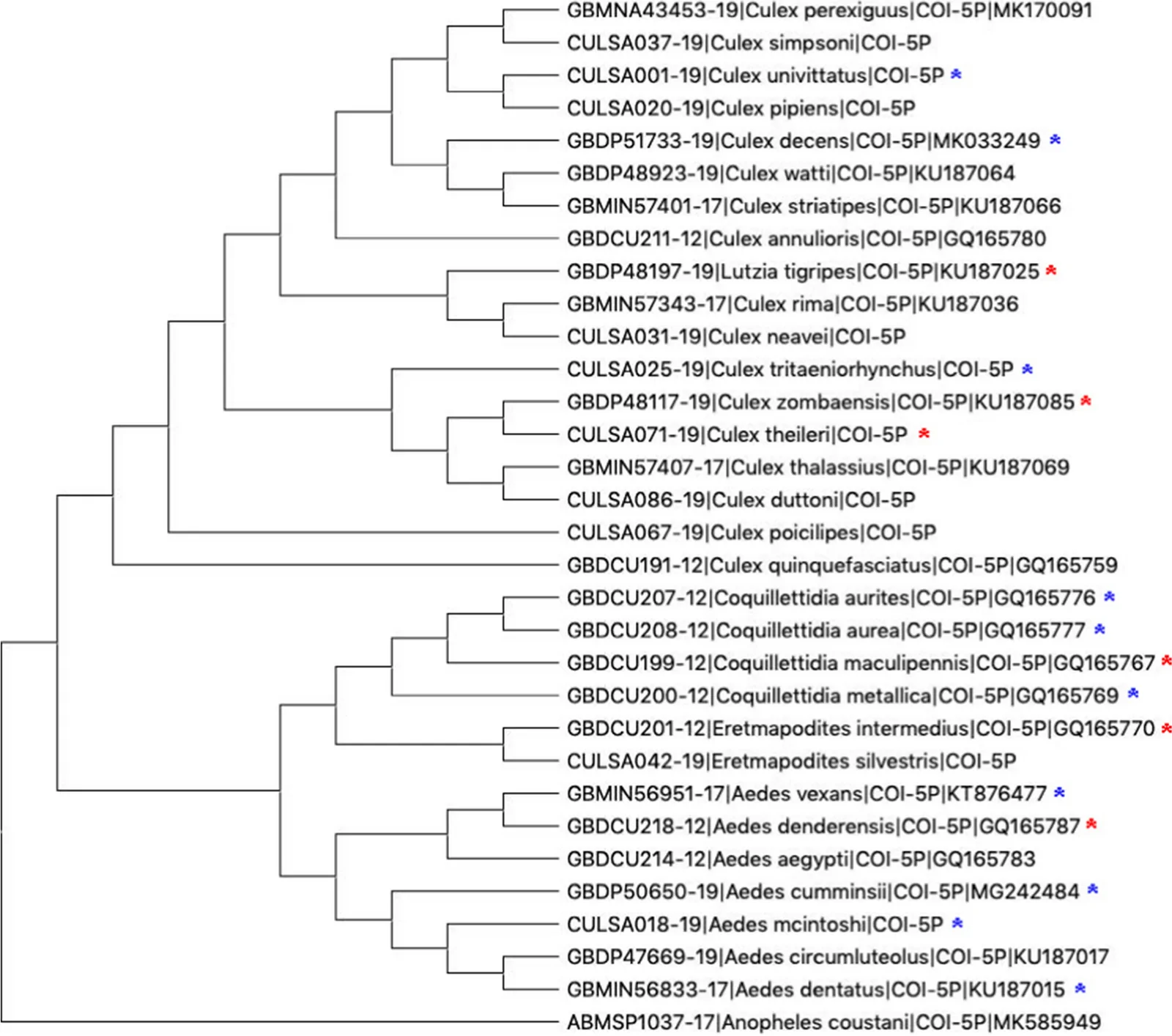
Phylogenetic tree of mosquito species found at Rwandan sampling sites created with publicly available high-quality and long-length cytochrome c oxidase subunit I (COI) sequences from BOLD derived from primarily Ugandan, Kenyan, and South African mosquitoes. Sequences were aligned and the tree was created using the neighbor-joining method with 100 Maximum Likelihood bootstrap replicates in MEGA X [30]. Asterisks denote Rwandan species captured in this project that carried *Wolbachia* spp., with the blue asterisks indicating between 0.5 and 100 average sequences and red asterisks indicating > 100 average sequences

*Wolbachia*, *Asaia*, and *Serratia* are genera of bacteria that have been implicated in controlling transmission of some arboviruses and parasites [29, 1, 15, 35, 39, 56, 58], and mosquito genera differed in beta diversity profiles with this component of the microbiome (Fig. 5). All *Coquillettidia* species caught in Rwanda were strong carriers of *Wolbachia* (Fig. 6)*. Aedes*, *Culex*, *Eretmapodites*, and *Lutzia* all had some species that carried *Wolbachia*.

The average number of sequences for each of the bacterial genera of interest varied widely by mosquito host species. Overall, the Nyabugogo disturbed site, which was close to human habitation, had higher numbers of average sequences of *Asaia*, *Serratia*, and *Wolbachia* than the preserved and heavily forested Huye campus site (Table 2). Interestingly, some mosquito host species that were found in abundance at both sampling locations had considerably different average numbers of sequences. For example, *Lutzia tigripes* was more relatively abundant at the Nyabugogo disturbed site and also had substantially higher average sequences of *Asaia* and *Wolbachia* at that same site. *Culex striatipes* (domestic) and *Culex rima* (sylvatic)*,* which were also more abundant at the Nyabugogo disturbed site, had substantially higher average *Asaia* and *Serratia* sequences compared to the Huye campus site as well. *Culex theileri*, a sylvatic mosquito, also had higher average sequences of *Serratia* and *Wolbachia* at the Nyabugogo disturbed site. In contrast, *Aedes mcintoshi* (ubiquitous) was found at both sites and was much more abundant at the Huye campus site, and had higher average sequences of *Asaia* and *Serratia* (but not *Wolbachia*) at the Huye campus site.

**Table 2:**
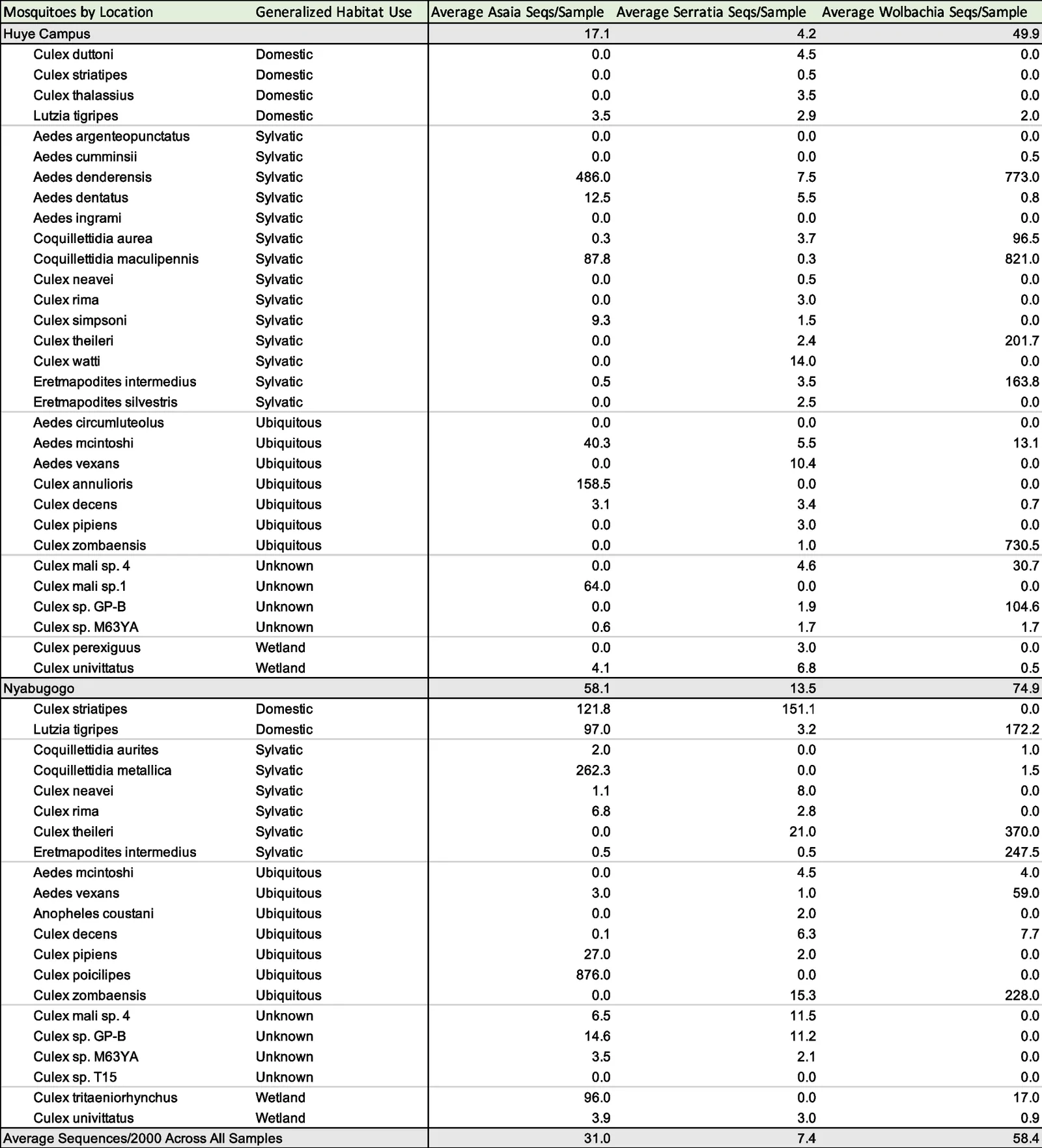
Average number of sequences for mosquito microbiome members of arboviral control interest (*Asaia, Serratia*, and *Wolbachia*). Mosquito species are separated by sampling location and are designated with their general habitat use classification as determined in Supplementary information references. These average sequence values are based on samples rarefied to 2000 sequences per sample

## Discussion

Rwandan mosquito microbiomes remain understudied and have the potential to provide imperative information in the regional fight against arboviral and *Plasmodium* infections. Here, we present the first study in Rwanda and one of the very few studies in the broader East African region analyzing the microbial assemblages present in communities of field-collected mosquitoes [43, 57]. The two sites studied here provide a survey of mosquito and microbial community assemblages in both disturbed, human-occupied habitat (Nyabugogo disturbed), and preserved, natural second-growth forest habitat (Huye campus). To best understand how to control arboviral and mosquito-borne parasite transmission using microbial control methods, it is essential to understand both domestic and sylvatic mosquito systems (Table 2). We focused on mosquito gut microbiomes. Previous studies of insects indicated that the abundance of microbes in the gut vastly overwhelms the surface microbiome signal [22]. Surface microbiomes are not necessarily environmental contaminants but could be important physiologically [60].

While the two sampling sites did not have significant differences in microbial communities and were composed of a mixture of mosquito species preferring habitats from sylvatic to wetland to domestic, a broader survey of sites is needed to determine the scale at which both mosquito species and microbiomes are environmentally impacted. Additional studies to build our understanding of naturally formed microbial communities are essential to safely employing microbial control methods and further reducing the burden of mosquito-borne diseases.

In terms of alpha diversity metrics (Observed sOTU richness and the Shannon Diversity Index), we did not see any strong trends or differences between groups, including genera, species, and sampling location. We hypothesize that this lack of differences between genera, species, and locations may be due to similar habitable area and selection pressures in the internal environment of the mosquitoes, though further work must be undertaken to address this. Differences in alpha diversity between visibly blood-fed and non-blood-fed mosquitoes were anticipated based on prior studies regarding the reducing environment in the mosquito gut following blood-feeding by females [37, 57, 29]. However, our data showed a weak difference between these groups. This is likely due to the visual nature of confirming blood-fed status of females in this study. Only female mosquitoes that were visibly engorged upon capture were considered blood-fed, which does not include females that had previously fed but digested the blood and were no longer engorged.

In terms of beta diversity metrics (Jaccard and Weighted Unifrac distances), several interesting trends emerged in the data. Genera, species, and location were all highly significantly different, demonstrating that taxa and broader habitat play a key role in the community assemblage of the mosquito microbiome under field conditions. The lack of difference between catch methods indicated that there was not a sampling bias based on the method of capture utilized for mosquitoes. The absence of difference in beta diversity for blood-fed status indicates that, similarly to our alpha diversity measures, recently fed females that had digested blood were probable in the non-blood-fed group. Mosquito sexes were not different in terms of microbial community assemblage, though they were anticipated to be different based on differences in feeding between sexes. Here, we hypothesize that environmental drivers and mosquito microhabitat use are more important than sex in terms of the formation of internal microbial communities, though this will also require additional investigation to better understand these dynamics. Several previous studies demonstrated differences in gut microbiomes among male and female mosquitoes [33, 34].

The core microbiome, or the 20 most abundant sOTUs appearing in nearly all samples in high relative abundance, included numerous members well documented as being abundant in mosquito microbiomes in other studies around the world [5, 11, 16, 18, 39, 43, 57]. *Asaia*, *Serratia*, and *Wolbachia* are genera of bacteria of particular interest for controlling arboviral transmission, and all three were well represented in the core microbiomes of the mosquitoes collected in this study. The average abundance of these genera was much higher at the Nyabugogo disturbed site than at the Huye campus site. The distribution of mosquitoes carrying these microbes was not continuous across any given genus, but rather varied greatly by species. Several mosquitoes that appeared in abundance at both sites had much higher average sequences of these bacteria at the Nyabugogo disturbed site than at the Huye campus site. Our results also reflect previous negative associations between *Asaia* and *Wolbachia* in individual mosquitoes [26, 49]. While these interactions are inherently complex, we suggest that the likelihood of symbiosis with these microbes of interest indicates that microbial establishment in mosquito hosts is strongly impacted by microhabitat use.

In order to more fully understand mosquito microbial interactions in situ, studies further examining the level of influence that specific environmental factors, such as temperature and rainfall, have over the formation of symbioses between microbes and mosquitoes. These studies should be undertaken both in a laboratory and field setting, as controlling variables in the field can present challenges, whereas work in the lab does not fully realize all potential variables influencing microbe-mosquito symbiosis. We would also recommend pursuing additional studies on the eukaryotic, fungal, and viral members of the mosquito microbiome for a more complete picture of these microbial communities in a field setting. Studies of the natural microbiota are critical for disease mitigation measures that seek to manipulate microbial taxa because some communities are more refractory than others and may impede *Wolbachia* or other beneficial species [26].

Our findings help to expand on our understanding of wild mosquito microbiomes in an area of the world that is particularly at risk for high arboviral transmission. In order to safely utilize microbial tools for mosquito control, such as *Wolbachia*-infected mosquito releases, we must first understand the natural communities of both mosquito hosts and microbial inhabitants and the factors that influence these interactions.

## Supplementary Information

Below is the link to the electronic supplementary material.

Supplementary file1 (DOCX 148 KB)

## Data Availability

**[Upon publication]** The datasets generated and analyzed during the current study are publicly available. This data can be found here: http://www.ncbi.nlm.nih.gov/bioproject/849234.
