# Supplementary material for: Mosquito Microbiomes of Rwanda: Characterizing Mosquito Host and Microbial Communities in the Land of a Thousand Hills
Source: Microb Ecol. 2024 May 1;87:64. doi: 10.1007/s00248-024-02382-3 (PMC11062966; doi:10.1007/s00248-024-02382-3)
Supplement: Supplementary file 1 [file 248_2024_2382_MOESM1_ESM.docx]

**Supplementary Information**

1. Supplementary references that indicate the human pathogens and parasites that each genus is capable of transmitting. This list is not exhaustive and is summarized in Table 1.

Ajamma, Y. U., Onchuru, T. O., Ouso, D. O., Omondi, D., Masiga, D. K., & Villinger, J. (2018). Vertical transmission of naturally occurring Bunyamwera and insect-specific flavivirus infections in mosquitoes from islands and mainland shores of Lakes Victoria and Baringo in Kenya. *PLoS Neglected Tropical Diseases*, *12*(11). https://doi.org/10.1371/journal.pntd.0006949

Appawu, M. A., Dadzie, S. K., & Quartey, S. Q. (2000). Studies on the feeding behaviour of larvae of the predaceous mosquito *Culex* (*Lutzia*) *tigripes* Grandpre and Chamoy (Diptera: Culicidae). *Insect Science and Its Application*, *20*(4), 245–250. https://doi.org/10.1017/S1742758400015599

Arum, S. O., Weldon, C. W., Orindi, B., Landmann, T., Tchouassi, D. P., Affognon, H. D., & Sang, R. (2015). Distribution and diversity of the vectors of Rift Valley fever along the livestock movement routes in the northeastern and coastal regions of Kenya. *Parasites and Vectors*, *8*(1), 294. https://doi.org/10.1186/s13071-015-0907-1

Becker, N., Petrić, D., Zgomba, M., Boase, C., Madon, M., Dahl, C., & Kaiser, A. (2010). Mosquitoes and their control: Second edition. In *Mosquitoes and Their Control: Second Edition*. Springer-Verlag Berlin Heidelberg. https://doi.org/10.1007/978-3-540-92874-4

Benelli, G., Bedini, S., Cosci, F., Toniolo, C., Conti, B., & Nicoletti, M. (2015). Larvicidal and ovideterrent properties of neem oil and fractions against the filariasis vector *Aedes albopictus* (Diptera: Culicidae): a bioactivity survey across production sites. *Parasitology Research*, *114*(1), 227–236. https://doi.org/10.1007/s00436-014-4183-3

Biteye, B., Fall, A. G., Seck, M. T., Ciss, M., Diop, M., & Gimonneau, G. (2019). Host-feeding patterns of *Aede*s (*Aedimorphus*) *vexans arabiensis*, a Rift Valley Fever virus vector in the Ferlo pastoral ecosystem of Senegal. *PLoS ONE*, *14*(10). https://doi.org/10.1371/journal.pone.0215194

Cansado-Utrilla, C., Jeffries, C. L., Kristan, M., Brugman, V. A., Heard, P., Camara, G., Sylla, M., Beavogui, A. H., Messenger, L. A., & Walker, T. (2019). An assessment of adult mosquito collection techniques for studying species abundance and diversity in Maferinyah, Guinea. *BioRxiv*, 772822. https://doi.org/10.1101/772822

Blackmore, C. G. M., Blackmore, M. S., Grimstad, P. R. (1998). Role of Anopheles *Quadrimaculatus* and *Coquillettidia* *perturbans* (Diptera: Culicidae) in the Transmission Cycle of Cache Valley Virus (Bunyaviridae: Bunyavirus) in the Midwest, USA. *Journal of Medical Entomology*, *35*(5). https://doi.org/10.1093/JMEDENT/35.5.660

Corbet, P. S., Williams, M. C., & Gillett, J. D. (1961). O’nyong-nyong fever: An epidemic virus disease in East Africa. IV. Vector studies at epidemic sites. *Transactions of the Royal Society of Tropical Medicine and Hygiene*, *55*(5), 463–480. https://doi.org/10.1016/0035-9203(61)90095-5

*Culicidae Classification | Mosquito Taxonomic Inventory*. (n.d.). Retrieved May 30, 2020, from http://mosquito-taxonomic-inventory.info/simpletaxonomy/term/6090

Devi, B., Noren Singh, S., Chingangbam Singh, D., Salam Noren Singh, C., & Devi Moirangthem, B. (2018). *Lutzia tigripes* (Diptera: Culicidae, Metalutzia) for the mosquito larval control: A new prospect of mosquito control. *International Journal of Mosquito Research*, *5*(6), 1–04.

Diallo, D., Talla, C., Ba, Y., Dia, I., Sall, A. A., & Diallo, M. (2011). Temporal distribution and spatial pattern of abundance of the Rift Valley fever and West Nile fever vectors in Barkedji, Senegal. *Journal of Vector Ecology*, *36*(2), 426–436. https://doi.org/10.1111/j.1948-7134.2011.00184.x

Dohm, D. J., Logan, T. M., Barth, J. F., & Turell, M. J. (1995). Laboratory Transmission of Sindbis Virus by *Aedes albopictus, Ae. aegypti*, and *Culex pipiens* (Diptera: Culicidae). *Journal of Medical Entomology*, *32*(6), 818–821. https://doi.org/10.1093/jmedent/32.6.818

Faran, M. E., Turell, M. J., Romoser, W. S., Routier, R. G., Gibbs, P. H., Cannon, T. L., & Bailey, C. L. (1987). Reduced survival of adult *Culex pipiens* infected with Rift Valley fever virus. *American Journal of Tropical Medicine and Hygiene*, *37*(2), 403–409. https://doi.org/10.4269/ajtmh.1987.37.403

Fontenille, D., & Jupp, P. G. (1989). [The presence of the *Culex* (*Culex*) *neavei* mosquito in Madagascar, its relevance in the transmission of arboviruses]. *Archives de l’Institut Pasteur de Madagascar*, *56*(1), 287–295.

Fontenille, D., Traore-Lamizana, M., Diallo, M., Thonnon, J., Digoutte, J. P., & Zeller, H. G. (1998). New vectors of Rift Valley fever in West Africa. In *Emerging Infectious Diseases* (Vol. 4, Issue 2, pp. 289–293). Centers for Disease Control and Prevention (CDC). https://doi.org/10.3201/eid0402.980218

Haddow, A. D., Nasar, F., Guzman, H., Ponlawat, A., Jarman, R. G., Tesh, R. B., & Weaver, S. C. (2016). Genetic Characterization of Spondweni and Zika Viruses and Susceptibility of Geographically Distinct Strains of *Aedes aegypti, Aedes albopictus* and *Culex quinquefasciatus* (Diptera: Culicidae) to Spondweni Virus. *PLoS Neglected Tropical Diseases*, *10*(10). https://doi.org/10.1371/journal.pntd.0005083

Haddow, A. J. (1946). The mosquitoes of Bwamba County, Uganda: IV.—Studies on the genus *Eretmapodites*, Theobald. *Bulletin of Entomological Research*, *37*(1), 57–82. https://doi.org/10.1017/S0007485300021994

Hamer, G. L., Kitron, U. D., Brawn, J. D., Loss, S. R., Ruiz, M. O., Goldberg, T. L., & Walker, E. D. (2008). *Culex pipiens* (Diptera: Culicidae): A Bridge Vector of West Nile Virus to Humans. *Journal of Medical Entomology*, *45*(1), 125–128. https://doi.org/10.1093/jmedent/45.1.125

Hylton, A. R. (1969). Studies on Longevity of Adult *Eretmapodites chrysogaster, Aedes togoi and Aedes* (*Stegomyia*) *albopictus* Females (Diptera: Culicidae). *Journal of Medical Entomology*, *6*(2), 147–149. https://doi.org/10.1093/jmedent/6.2.147

Jeffery, J. AL, Ryan, P. A., Lyons, S. A., & Kay, B. H. (2002). Vector competence of *Coquillettidia linealis* (Skuse) (Diptera: Culicidae) for Ross River and Barmah Forest viruses. *Australian Journal of Entomology*, *41*(4), 339–344. https://doi.org/10.1046/j.1440-6055.2002.00316.x

Jupp, P. G., & McIntosh, B. M. (1970). Quantitative Experiments on the Vector Capability of Theobald with West Nile and Sindbis Viruses. *Journal of Medical Entomology*, *7*(3), 371–373. https://doi.org/10.1093/JMEDENT/7.3.371

Karungu, S., Atoni, E., Ogalo, J., Mwaliko, C., Agwanda, B., Yuan, Z., & Hu, X. (2019). Mosquitoes of Etiological Concern in Kenya and Possible Control Strategies. *Insects*, *10*(6), 173. https://doi.org/10.3390/insects10060173

Kilpatrick, A. M., Kramer, L. D., Campbell, S. R., Alleyne, E. O., Dobson, A. P., & Daszak, P. (2005). West Nile virus risk assessment and the bridge vector paradigm. *Emerging Infectious Diseases*, *11*(3), 425–429. https://doi.org/10.3201/eid1103.040364

Kovendan, K., Murugan, K., & Vincent, S. (2012). Evaluation of larvicidal activity of *Acalypha alnifolia* Klein ex Willd. (Euphorbiaceae) leaf extract against the malarial vector, *Anopheles stephensi*, dengue vector, *Aedes aegypti* and *Bancroftian filariasis* vector, *Culex quinquefasciatus* (Diptera: Culicidae). *Parasitology Research*, *110*(2), 571–581. https://doi.org/10.1007/s00436-011-2525-y

LaBeaud, A. D., Sutherland, L. J., Muiruri, S., Muchiri, E. M., Gray, L. R., Zimmerman, P. A., Hise, A. G., & King, C. H. (2011). Arbovirus prevalence in mosquitoes, Kenya. *Emerging Infectious Diseases*, *17*(2), 233–241. https://doi.org/10.3201/eid1702.091666

Lutomiah, J., Omondi, D., Masiga, D., Mutai, C., Mireji, P. O., Ongus, J., Linthicum, K. J., & Sang, R. (2014). Blood meal analysis and virus detection in blood-fed mosquitoes collected during the 2006-2007 rift valley fever outbreak in Kenya. *Vector-Borne and Zoonotic Diseases*, *14*(9), 656–664. https://doi.org/10.1089/vbz.2013.1564

Lutomiah, J., Ongus, J., Linthicum, K. J., & Sang, R. (2014). Natural Vertical Transmission of Ndumu Virus in *Culex pipiens* (Diptera: Culicidae) Mosquitoes Collected as Larvae . *Journal of Medical Entomology*, *51*(5), 1091–1095. https://doi.org/10.1603/me14064

Mangiafico, J. A. (1971). Chikungunya virus infection and transmission in five species of mosquito. *The American Journal of Tropical Medicine and Hygiene*, *20*(4), 642–645. https://doi.org/10.4269/ajtmh.1971.20.642

McGreevy, P. B., McGreevy, P. B., Theis, J. H., & Clark, J. (1974). Studies on Filariasis. III. *Dirofilaria immitis*: Emergence of Infective Larvae from the Mouthparts of *Aedes aegypti*. *Journal of Helminthology*, *48*(4), 221–228. https://doi.org/10.1017/S0022149X00022896

Moncayo, A. C., Edman, J. D., & Turell, M. J. (2000). Effect of Eastern Equine Encephalomyelitis Virus on the Survival of *Aedes albopictus, Anopheles quadrimaculatus*, and *Coquillettidia perturbans* (Diptera: Culicidae). *Journal of Medical Entomology*, *37*(5), 701–706. https://doi.org/10.1603/0022-2585-37.5.701

Musa, A. A., Muturi, M. W., Musyoki, A. M., Ouso, D. O., Oundo, J. W., Makhulu, E. E., Wambua, L., Villinger, J., & Jeneby, M. M. (2020). Arboviruses and Blood Meal Sources in Zoophilic Mosquitoes at Human-Wildlife Interfaces in Kenya. *Vector-Borne and Zoonotic Diseases*, vbz.2019.2563. https://doi.org/10.1089/vbz.2019.2563

Mutebi, J.-P., Crabtree, M. B., Kading, R. C., Powers, A. M., Lutwama, J. J., & Miller, B. R. (2013). Mosquitoes of Western Uganda. *Journal of Medical Entomology*, *49*(6), 1289–1306. https://doi.org/10.1603/me12111

Ndiaye, E. H., Fall, G., Gaye, A., Bob, N. S., Talla, C., Diagne, C. T., Diallo, D., Ba, Y., Dia, I., Kohl, A., Sall, A. A., & Diallo, M. (2016). Vector competence of *Aedes vexans* (*Meigen*), *Culex poicilipes* (Theobald) and *Cx. quinquefasciatus* Say from Senegal for West and East African lineages of Rift Valley fever virus. *Parasites and Vectors*, *9*(1), 94. https://doi.org/10.1186/s13071-016-1383-y

Nikolay, B., Diallo, M., Faye, O., Boye, C. S., & Sall, A. A. (2012). Vector competence of *Culex neavei* (Diptera: Culicidae) for Usutu virus. *American Journal of Tropical Medicine and Hygiene*, *86*(6), 993–996. https://doi.org/10.4269/ajtmh.2012.11-0509

Njabo, K. Y., Cornel, A. J., Sehgal, R. N. M., Loiseau, C., Buermann, W., Harrigan, R. J., Pollinger, J., Valkiunas, G., & Smith, T. B. (2009a). Coquillettidia (Culicidae, Diptera) mosquitoes are natural vectors of avian malaria in Africa. *Malaria Journal*, *8*(1), 1–12. https://doi.org/10.1186/1475-2875-8-193

Njabo, K. Y., Cornel, A. J., Sehgal, R. N. M., Loiseau, C., Buermann, W., Harrigan, R. J., Pollinger, J., Valkiunas, G., & Smith, T. B. (2009b). *Coquillettidia* (Culicidae, Diptera) mosquitoes are natural vectors of avian malaria in Africa. *Malaria Journal*, *8*(1), 193. https://doi.org/10.1186/1475-2875-8-193

Pullan, R. L., Bukirwa, H., Staedke, S. G., Snow, R. W., & Brooker, S. (2010). *Plasmodium* infection and its risk factors in eastern Uganda. *Malaria Journal*, *9*(1), 2–2. https://doi.org/10.1186/1475-2875-9-2

Sardelis, M. R., Turell, M. J., Dohm, D. J., & O’Guinn, M. L. (2001). Vector competence of selected North American *Culex* and *Coquillettidia* mosquitoes for West Nile virus. *Emerging Infectious Diseases*, *7*(6), 1018–1022. https://doi.org/10.3201/eid0706.010617

Sharma, R. S., Kaul, S. M., & Sokhay, J. (2005). Seasonal fluctuations of dengue fever vector, *Aedes aegypti* (Diptera: Culicidae) in Delhi, India. *Southeast Asian Journal of Tropical Medicine and Public Health*, *36*(1), 186–190.

Smithburn, K.C., Haddow, A.J., Lumsden, W.H.R. (1949). Rift Valley fever; transmission of the virus by mosquitoes. *British Journal of Experimental Pathology*, *30*(1), 35–47.

Snow, W. F., & Boreham, P. F. L. (1978). The host-feeding patterns of some Culicine mosquitoes (Diptera: Culicidae) in the Gambia. *Bulletin of Entomological Research*, *68*(4), 695–706. https://doi.org/10.1017/S0007485300009652

Traore-Lamizana, M., Zeller, H. G., Mondo, M., Hervy, J.-P., Adam, F., & Digoutte, J.-P. (1994). Isolations of West Nile and Bagaza Viruses from Mosquitoes (Diptera: Culicidae) in Central Senegal (Ferlo). *Journal of Medical Entomology*, *31*(6), 934–938. https://doi.org/10.1093/jmedent/31.6.934

Valkiunas, G., Iezhova, T. A., Loiseau, C., Chasar, A., Smith, T. B., & Sehgal, R. N. M. (2008). New species of haemosporidian parasites (Haemosporida) from African rainforest birds, with remarks on their classification. *Parasitology Research*, *103*(5), 1213–1228. https://doi.org/10.1007/s00436-008-1118-x

Waddell, L., Pachal, N., Mascarenhas, M., Greig, J., Harding, S., Young, I., & Wilhelm, B. (2019). Cache Valley virus: A scoping review of the global evidence. *Zoonoses and Public Health*, *66*(7), 739–758. https://doi.org/10.1111/zph.12621

2. Supplementary references that indicate the general habitat use classification (Domestic, Sylvatic, Ubiquitous, Unknown, or Wetland) for each species of mosquito identified in Rwanda. These classifications are summarized in Table 2.

Burke, R., Barrera, R., Lewis, M., Kluchinsky, T., & Claborn, D. (2010). Septic tanks as larval habitats for the mosquitoes *Aedes aegypti* and *Culex quinquefasciatus* in Playa-Playita, Puerto Rico. *Medical and Veterinary Entomology*, *24*(2), 117–123. <https://doi.org/10.1111/j.1365-2915.2010.00864.x>

Chabaud, M. A., & Ovazza, M. (1958). [Yellow fever in the Federation of Ethiopia & Eritrea; present-day epidemiological data]. *Bulletin of the World Health Organization*, *19*(1), 7–21.

Diallo, D., Diagne, C. T., Hanley, K. A., Sall, A. A., Buenemann, M., Ba, Y., Dia, I., Weaver, S. C., & Diallo, M. (2012). Larval ecology of mosquitoes in sylvatic arbovirus foci in southeastern Senegal. *Parasites and Vectors*, *5*(1), 286. https://doi.org/10.1186/1756-3305-5-286

Gibbins, E. G. (1942). On the habits and breeding-places of *Aëdes* (*Stegomyia*) *simpsoni* Theobald in Uganda. *Annals of Tropical Medicine and Parasitology*, *36*(4), 151–160. https://doi.org/10.1080/00034983.1942.11685150

Haddow, A. J. (1956). Observations on the biting-habits of African mosquitos in the genus *Eretmapodites* Theobald. *Bulletin of Entomological Research*, *46*(4), 761–772. https://doi.org/10.1017/S0007485300037020

Junglen, S., Kurth, A., Kuehl, H., Quan, P. L., Ellerbrok, H., Pauli, G., Nitsche, A., Nunn, C., Rich, S. M., Lipkin, W. I., Briese, T., & Leendertz, F. H. (2009). Examining landscape factors influencing relative distribution of mosquito genera and frequency of virus infection. *EcoHealth*, *6*(2), 239–249. https://doi.org/10.1007/s10393-009-0260-y

Lu, S., Chourey, K., Reiche, M., Nietzsche, S., Shah, M. B., Neu, T. R., Hettich, R. L., & Küsel, K. (2013). Insights into the structure and metabolic function of microbes that shape pelagic iron-rich aggregates. *Applied and Environmental Microbiology*, *79*(14), 4272–4281. https://doi.org/10.1128/AEM.00467-13

Lutomiah, J., Bast, J., Clark, J., Richardson, J., Yalwala, S., Oullo, D., Mutisya, J., Mulwa, F., Musila, L., Khamadi, S., Schnabel, D., Wurapa, E., & Sang, R. (2013). Abundance, diversity, and distribution of mosquito vectors in selected ecological regions of Kenya: public health implications. *Journal of Vector Ecology*, *38*(1), 134–142. https://doi.org/10.1111/j.1948-7134.2013.12019.x

Miller, B. R., Nasci, R. S., Godsey, M. S., Savage, H. M., Lutwama, J. J., Lanciotti, R. S., & Peters, C. J. (2000). First field evidence for natural vertical transmission of West Nile virus in *Culex univittatus* complex mosquitoes from Rift Valley Province, Kenya. *American Journal of Tropical Medicine and Hygiene*, *62*(2), 240–246. https://doi.org/10.4269/ajtmh.2000.62.240

Mutebi, J.-P., Crabtree, M. B., Kading, R. C., Powers, A. M., Lutwama, J. J., & Miller, B. R. (2013). Mosquitoes of Western Uganda. *Journal of Medical Entomology*, *49*(6), 1289–1306. https://doi.org/10.1603/me12111

Muturi, E. J., Shililu, J. I., Gu, W., Jacob, B. G., Githure, J. I., & Novak, R. J. (2007). Larval habitat dynamics and diversity of *Culex* mosquitoes in rice agro-ecosystem in Mwea, Kenya. *American Journal of Tropical Medicine and Hygiene*, *76*(1), 95–102. <https://doi.org/10.4269/ajtmh.2007.76.95>

Muturi, E. J., Shililu, J., Jacob, B., Gu, W., Githure, J., & Novak, R. (2006). Mosquito species diversity and abundance in relation to land use in a riceland agroecosystem in Mwea, Kenya. *Journal of Vector Ecology*, *31*(1), 129–137. https://doi.org/10.3376/1081-1710(2006)31[129:msdaai]2.0.co;2

Ndiaye, E. H., Fall, G., Gaye, A., Bob, N. S., Talla, C., Diagne, C. T., Diallo, D., Ba, Y., Dia, I., Kohl, A., Sall, A. A., & Diallo, M. (2016). Vector competence of *Aedes vexans* (*Meigen*), *Culex poicilipes* (Theobald) and *Cx. quinquefasciatus* Say from Senegal for West and East African lineages of Rift Valley fever virus. *Parasites and Vectors*, *9*(1), 94. https://doi.org/10.1186/s13071-016-1383-y

Obame-Nkoghe, J., Rahola, N., Ayala, D., Yangari, P., Jiolle, D., Allene, X., Bourgarel, M., Maganga, G. D., Berthet, N., Leroy, E. M., & Paupy, C. (2017). Exploring the diversity of bloodsucking Diptera in caves of Central Africa. *Scientific Reports*, *7*(1), 1–11. <https://doi.org/10.1038/s41598-017-00328-z>

Robert, V., Awono-Ambene, H. P., & Thioulouse, J. (1998). Ecology of Larval Mosquitoes, with Special Reference to Anopheles arabiensis (Diptera: Culcidae) in Market-Garden Wells in Urban Dakar, Senegal. *Journal of Medical Entomology*, *35*(6), 948–955. https://doi.org/10.1093/jmedent/35.6.948

Roiz, D., Ruiz, S., Soriguer, R., & Figuerola, J. (2015). Landscape effects on the presence, abundance and diversity of mosquitoes in mediterranean wetlands. *PLoS ONE*, *10*(6). <https://doi.org/10.1371/journal.pone.0128112>

Sallam, M. F., Al Ahmed, A. M., Abdel-Dayem, M. S., & Abdullah, M. A. R. (2013). Ecological Niche Modeling and Land Cover Risk Areas for Rift Valley Fever Vector, *Culex tritaeniorhynchus* Giles in Jazan, Saudi Arabia. *PLoS ONE*, *8*(6), e65786. https://doi.org/10.1371/journal.pone.0065786

Self, L. S., Shin, H. K., Kim, K. H., Lee, K. W., Chow, C. Y., & Hong, H. K. (1973). Ecological studies on *Culex tritaeniorhynchus* as a vector of Japanese encephalitis. *Bulletin of the World Health Organization*, *49*(1), 41–47.

Snow, W. F. (1987). Studies of house-entering habits of mosquitoes in The Gambia, West Africa: experiments with prefabricated huts with varied wall apertures. *Medical and Veterinary Entomology*, *1*(1), 9–21. https://doi.org/10.1111/j.1365-2915.1987.tb00318.x

Soti, V., Tran, A., Degenne, P., Chevalier, V., Lo Seen, D., Thiongane, Y., Diallo, M., Guégan, J. F., & Fontenille, D. (2012). Combining Hydrology and Mosquito Population Models to Identify the Drivers of Rift Valley Fever Emergence in Semi-Arid Regions of West Africa. *PLoS Neglected Tropical Diseases*, *6*(8). https://doi.org/10.1371/journal.pntd.0001795

Zahouli, J. B. Z., Koudou, B. G., Müller, P., Malone, D., Tano, Y., & Utzinger, J. (2017). Urbanization is a main driver for the larval ecology of *Aedes* mosquitoes in arbovirus-endemic settings in south-eastern Côte d’Ivoire. *PLoS Neglected Tropical Diseases*, *11*(7), e0005751. <https://doi.org/10.1371/journal.pntd.0005751>

**Supplementary Figure**

**Supplementary Figure S1.** Principle coordinate analysis based on weighted Unifrac distances to demonstrate the relative community composition of the microbiome for each species by sampling location. Some species were only found at one location and therefore do not show both sites in the figure.
